# 

**Published:** 1899-09

**Authors:** 


### PERSONALS.

Dr. G. K. Swank, of East Mauch Chunk, was a visitor to Philadelphia in August.

Dr. Leonard Pearson, of Philadelphia, was a sojourner for a short period in August at Old Point Comfort, Va.

The visit of Dr. William Dougherty, of Baltimore, Md., to Boston was cut short by a severe and painful attack of gout, which we are glad to report has disappeared since his return to the "Old Line State."

Dr. Thomas B. Rogers, of Woodbury, N. J., reports the outbreak of anthrax in Gloucester County under control, but fears its reappearance next season.

Dr. Leonard Pearson, State Veterinarian, and J. T. Rothrock, Forestry Commissioner, are making a thorough investigation of the serious losses among the cattle of Carbon County, Pa., ostensibly due to some local cause of a vegetative character. A full report is promised later on. Some fifty deaths have occurred—all of a sudden nature.

Dr. W. Horace Hoskins, of the JOURNAL, was a recent visitor to Williamsport. His interest is centred in "Farmer" Creasy for State Treasurer; hence his mission.

Dr. R. S. Huidekoper, of the JOURNAL, still sojourns in rural Chester County, Pa., with an occasional visit to the "City of Brotherly Love."

Dr. Edward M. Ranck, of Philadelphia, Pa., shakes the dust of the city from his feet, the latter part of each week, for a rest and short sojourn in his native county of Lancaster—"the farmers' paradise" of Pennsylvania.

Dr. Charles H. Higgins, of Fitchburg, Mass., has gone to Montreal, Canada, for a protracted stay.

After almost fifteen years' army veterinary service, Dr. William J. Waugh has resigned to enter civil practice. In the recent examinations Dr. Waugh obtained a second-class rating, and his regiment was ordered to the Philippine Islands. The expense of maintaining a wife and three children in the United States on the meagre salary of a second veterinarianship, and those imposed at that far-away point, compelled his resignation. Such are the amenities of the United States Army Veterinary Service. Nations are many times ungrateful to those who serve them best.

Dr. S. L. Blount, of Temple, Texas, was a visitor to Philadelphia in August.

Dr. Henry Bowers, of Philadelphia, who recently became a partner in one of the "Quaker City's" largest livery stables, has sold out his interest.

Dr. Henry Cady, of Gloversville, N. Y., is an admirer of the trotter and is the owner of several speedy ones.

Dr. J. E. Gardiner, of Hartford, Conn., is the owner of several fast trotters. As a breeder Dr. Gardiner has bred and raised several fast ones.

Dr. Louis P. Cook, of the Bureau of Animal Industry, has been transferred from St. Louis, Missouri, to Cincinnati, Ohio. Dr. Cook has nearly four years' service to his credit, and was originally from Cincinnati.

Dr. M. H. Reynolds, of St. Anthony Park, Minnesota, has an excellent article in *The Farmer*, published at St. Paul, on "Tuberculosis and Tuberculin."

Dr. Harry Walters, formerly of Wilkesbarre, Pa., but now of Kirksville, Missouri, was a visitor to Philadelphia in the latter part of July. His impaired health has been completely restored by his sojourn in the West and relaxation from professional duties.

Dr. J. O. F. Price, of Lancaster, Ohio, has accepted a position as an assistant inspector in the Bureau of Animal Industry, and is stationed at Chicago, Illinois.

Dr. R. P. Lyman, of Hartford, Conn., dropped the exactions of professional work for a ten days' rest early in August.

Dr. H. S. Creighton, of Winfield, Kansas, conducts an infirmary in connection with his practice.

Dr. C. A. Spicer, of Pittsburg, a devotee of the wheel, has just won a lawsuit against one of the "Smoky City" cab companies for injuries done him and his bicycle.

City Veterinarian Snyder, of Cincinnati, Ohio, was a sojourner at Atlantic City, N. J., in August.

Dr. Emil Knight, of Rochester, N. Y., spent some ten days in August along the New Jersey coast seaside resorts. He was also a visitor to Philadelphia, and spent a day at the college of his graduation.

Dr. George B. Jobson, of Franklin, Pa., had an excellent article in a recent issue of the Oil City *Derrick*, reviewing the splendid work done by the Pennsylvania State Livestock Sanitary Board.

Dr. N. W. Drake, of Philadelphia, has been on the sick list from a severe attack of rheumatism.

Drs. W. A. Meredith, of Corry, Pa., and James Meredith, of Jamestown, N. Y., were visitors in Philadelphia in September, and attended the sessions of the convention in New York.

Dr. John W. Adams, of Philadelphia, spent a part of his vacation period in and around Buffalo, N. Y., during September.

Dr. A. G. Kern, of Knoxville, Tenn., was among those who viewed the "Dewey parade" in New York prior to his entering the Medical Department of the University of Pennsylvania.

Dr. and Mrs. H. D. Gill, of New York City, are among those who lead a fast pace among the roadster classes on the Speedway, and few are willing to try their speedy ones against them.

Dr. R. S. Huidekoper, late Chief Surgeon of the First Army Corps, will address the Convention of Military Surgeons in Kansas City in October on the status of the army medical service in our recent war with Spain.

Dr. N. P. Hinkley, of Buffalo, N. Y., is now a practitioner at Atlanta, Ga.

Dr. R. R. Bell, of Brooklyn, has become interested in a rubber horseshoe pad which possesses certain advantages over those now in the market, and which will shortly be placed in use in all large cities.

Dr. John J. Millar, City Veterinarian of Sioux City, Iowa, conducts a commodious veterinary hospital and kennel in connection with his practice.

Dr. Wm. R. Howe, of Dayton, Ohio, is now a resident of New York City, where he is connected with the "Expanding Tread Company of America," the proprietors of the "Rough Rider" horseshoe, consisting of a grooved drop-forged shoe, into which is inserted an expanding rubber band. Dr. Howe is the inventor of this shoe, and will devote his time to the introduction of it.

Dr. G. A. Johnston, of Sioux City, Iowa, was a visitor to Philadelphia and Washington subsequent to the meeting of the American Veterinary Medical Association.

Dr. H. D. Paxson, of the Bureau of Animal Industry, has been transferred to Kansas City, Kas., from Chicago.

Dr. R. D. Howard, of Castile, N. Y., carries on a drug store in connection with his veterinary practice.

Dr. Oscar Seeley, of Philadelphia, a graduate of the Veterinary Department of the University of Pennsylvania, was a visitor to the Virginia horseshows and fairs during the month of August.

Mr. L. A. Nolan, of Baltimore, Md., class of "1900," Veterinary Department of the University of Pennsylvania, was a visitor to the college and the JOURNAL office in August. He says the automobiles have been no barrier to large and lucrative returns to the "Oriole" city's veterinarians the past four months.

Dr. Wm. J. Tomlinson, of Williamsport, Pa., has one of the most complete hospital and stable plants in the Keystone State. Its construction and facilities exhibit the most thoughtful consideration of all the good features of such an institution.

Dr. Chas. H. Higgins, of Fitchburg, Mass., has accepted the position of pathologist to the Dominion of Canada Government in connection with the quarantine system, and will be located at Montreal.

Dr. A. T. Sedlers, of Camden, N. J., was on the sick list during the month of August.

Dr. E. B. Ackerman, of Brooklyn, looked occasionally upon practice during August, finding midsummer rustication more congenial.

Drs. Jobson and Swift, of Venango Co., Pa., dealt with a number of suspected cases of rabies in August, and sent several brains of animals to the State Live-stock Sanitary Board for examination and testing.

Drs. J. E. Ryder, R. W. Ellis, and F. R. Hanson, officiated as the committee having in charge the banquet and local arrangements of the silver anniversary of the American Veterinary College.

